# Efficacy of Single-Pill Combination of Telmisartan 80 mg and Hydrochlorothiazide 25 mg in Patients with Cardiovascular Disease Risk Factors: A Prospective Subgroup Analysis of a Randomized, Double-Blind, and Controlled Trial

**DOI:** 10.1155/2013/749830

**Published:** 2013-04-04

**Authors:** Harold Bays, Pingjin Gao, Birgit Völker, Michaela Mattheus, Luis M. Ruilope, Dingliang Zhu

**Affiliations:** ^1^Louisville Metabolic and Atherosclerosis Research Center Inc. (L-MARC), 3288 Illinois Avenue, Louisville, KY 40213, USA; ^2^Shanghai Ruijin Hospital, Shanghai 200025, China; ^3^Boehringer Ingelheim Pharma GmbH & Co. KG, 55216 Ingelheim, Germany; ^4^Hospital 12 de Octubre, 28041 Madrid, Spain

## Abstract

*Objective*. Report of prespecified and *post hoc* subgroup analyses of a randomized, controlled trial comparing telmisartan 80 mg/hydrochlorothiazide 25 mg (T80/H25) combination therapy with T80 monotherapy, according to the presence of cardiovascular disease (CVD) risk factors. *Methods*. Hypertensive patients were randomized (2 : 1) to receive T80/H25 or T80 for 6 weeks, following a 1-week, low-dose, and run-in period. Systolic blood pressure (SBP) and diastolic BP reductions and BP goal achievement were evaluated in patients with CVD risk factors: presence of diabetes mellitus (DM), renal impairment, increased body mass index (BMI), and 10-year estimated risk for coronary heart disease (CHD). *Results*. In total, 888 patients received treatment. Overall, T80/H25 therapy significantly reduced SBP more than T80 monotherapy, irrespective of patient subgroup. In patients with DM, renal impairment, high BMI, and high CHD risk, BP goal achievement rates (<140/90 mm Hg) at Week 7, among those treated with T80/H25, were 52.8%, 52.8%, 50.6%, and 38.5%, respectively. More patients with DM reached a guideline-based BP goal (<130/80 mm Hg) at 7 weeks with T80/H25 than with T80 monotherapy (16.7% versus 8.8%). Rates of treatment-related adverse events were low and comparable across patient subgroups. *Conclusions*. Antihypertensive treatment with T80/H25 single-pill combination is effective and generally well tolerated, irrespective of the presence of CVD risk factors.

## 1. Introduction

Hypertension commonly accompanies other cardiovascular disease (CVD) risk factors and comorbidities such as obesity, chronic kidney disease, diabetes mellitus (DM), and heart disease [1]. In these high CVD risk patients, early treatment of hypertension to attain blood pressure (BP) goals may be particularly important in order to help reduce CVD risk.

Around three quarters of patients with hypertension will require combination therapy in order to reach guideline-recommended BP goals [2, 3]. In high-risk hypertensive patients, the initial use of combination therapy may facilitate the achievement of BP goals [4] and help to lower the risk of target organ damage [2, 5–8]. This supports a strategy aimed at combining antihypertensive agents with complementary mechanisms of action [3, 9].

The combination of an angiotensin II receptor blocker (ARB) plus a thiazide diuretic is endorsed by international hypertension guidelines [4, 5]. As a result of evidence from the ONgoing Telmisartan Alone and in combination with Ramipril Global Endpoint Trial (ONTARGET) [10, 11], telmisartan is the only ARB currently approved for the reduction of CVD morbidity in patients with manifest atherothrombotic CVD (history of coronary heart disease (CHD), stroke, or peripheral arterial disease) or type 2 DM with documented target organ damage.

A prior multinational, double-blind, and double-dummy study demonstrated that initial treatment with the single-pill combination (SPC) of telmisartan 80 mg (T80) plus hydrochlorothiazide (HCTZ) 25 mg (H25) therapy in patients with grade 2 or 3 hypertension significantly reduced BP and produced higher BP goal attainment compared with T80 alone [12]. The large patient population in that study provided an opportunity for analysis of the response to telmisartan/HCTZ SPC compared with T80 monotherapy treatment within different subpopulations of patients with grade 2 or 3 hypertension.

This current analysis evaluated the efficacy and tolerability of SPC T80/H25 compared with T80 monotherapy in patients with CVD risk factors: presence of DM, renal impairment, increased body mass index (BMI), and 10-year risk score for CHD (based on tertiles), with additional *post hoc* analyses according to alternative guideline-recommended 10-year CHD risk groups [13].

## 2. Patients and Methods

### 2.1. Study Design

The trial was performed as a 7-week, multinational, phase IV, randomized, double-blind, active-controlled, parallel-group, and forced-titration study in patients with grade 2 or 3 hypertension. The trial was conducted between June 2009 and April 2010 (ClinicalTrials.gov identifier: NCT00926289). The study design was described in detail elsewhere [12]. In brief, after an open-label, placebo run-in treatment period of 1–14 days, patients were randomized 2 : 1 to double-blind treatment with SPC telmisartan 40 mg (T40)/HCTZ 12.5 mg or T40 monotherapy for 1 week before uptitration to the target dose of SPC T80/H25 or T80 monotherapy, respectively, for the remaining 6 weeks. The trial was conducted under the guidelines specified by the Declaration of Helsinki and International Conference on Harmonisation Tripartite Harmonised Guidelines for Good Clinical Practice. The study protocol was approved by the health authority in each country and by the institutional review board or ethics committee of each center. Study participants provided written informed consent.

### 2.2. Patients and Subgroups for Analysis

Patients were recruited at 102 participating centers in eight countries (Bulgaria, China, France, Georgia, Romania, Russia, South Korea, and the United States). Eligible patients were men or women age ≥18 years with grade 2 or 3 hypertension (mean seated in-clinic trough cuff systolic BP (SBP) ≥160 mm Hg and diastolic BP (DBP) ≥100 mm Hg) who met the inclusion criteria (described in detail elsewhere) [12]. Study exclusion criteria included mean SBP ≥200 mm Hg and/or DBP ≥120 mm Hg; severe renal impairment (serum creatinine >3.0 mg/dL and/or creatinine clearance <30 mL/min and/or clinical markers of severe renal impairment); congestive heart failure (New York Heart Association Functional Class III or IV); severe obstructive coronary artery disease; aortic stenosis; contraindications to a placebo run-in period (e.g., stroke within the past 6 months, myocardial infarction, cardiac surgery, percutaneous transluminal coronary angioplasty, unstable angina, or coronary artery bypass graft within 3 months prior to the start of the placebo run-in period); and uncontrolled DM (glycated hemoglobin ≥10%).

In this analysis, patients were evaluated for inclusion into baseline CVD risk factor subgroups: DM, renal function, BMI, and 10-year CHD risk score. The DM subgroup included those with a diagnosis of type 1 DM, type 2 DM, diabetic retinopathy/nephropathy, or the presence of recognized Medical Dictionary for Regulatory Activities codes for DM. Renal function categories were defined by estimated glomerular filtration rate (eGFR) <60 mL/min/1.73 m^2^ or eGFR ≥60 mL/min/1.73 m^2^. BMI categories were defined as <25 kg/m^2^, ≥25–<30 kg/m^2^, or ≥30 kg/m^2^.

For 10-year CHD risk score, the probability of developing CHD over 10 years was estimated (based on a risk score developed from the Framingham Heart Study) for all treated patients for whom a baseline laboratory value for total cholesterol and high-density lipoprotein (HDL) was available. This risk score estimated the probability of developing CHD over 10 years based on baseline values for age, gender, total cholesterol and HDL, BP category, presence of DM (yes/no), and smoking status (yes/no) [14]. The method used in this analysis to assess the 10-year risk for CHD was based upon a version of the Framingham Risk Score described by Wilson et al., which included DM as a measured parameter [14].

The prespecified analysis according to the trial statistical analysis plan divided CHD risk by tertiles across the patient population, which provided 10-year CHD risk cutoffs of <3.62%, ≥3.62–<8.66%, and ≥8.66%. An additional *post hoc* analysis assessed CHD risk according to more conventional and established categories of low, medium, and high CHD risk [13] defined as follows: CHD risk category 1 (CHD1) <10%, CHD risk category 2 (CHD2) 10%–<20%, and CHD risk category 3 (CHD3) ≥20%.

### 2.3. Efficacy and Safety Evaluations

At each study visit, seated trough cuff BP was measured approximately 24 hours (20–30 hours) after the last study drug intake, with the mean taken from three consecutive measurements performed approximately 2 minutes apart using a standard manual cuff sphygmomanometer or other validated device (with cuff size conforming with American Heart Association Guidelines) [15]. BP measurements were performed at screening, at the start of the open-label placebo run-in treatment period, at the end of the run-in treatment period prior to randomization (i.e., at baseline), and then after 1, 3, 5, and 7 weeks of double-blind treatment.

Efficacy endpoints assessed at Weeks 3, 5, and 7 were described previously [12] and included the primary endpoint measure of change from baseline to final visit (Week 7) in mean seated trough cuff SBP. Secondary and other endpoints included change from baseline to final visit (Week 7) in mean seated trough cuff DBP, the proportion of patients achieving overall BP goal (defined as a mean seated trough cuff SBP/DBP <140/90 mm Hg), the proportion of patients with DM achieving overall BP goal (defined as a mean seated trough cuff SBP/DBP <130/80 mm Hg), the proportion of patients achieving SBP goal (mean SBP <140 mm Hg), the proportion of patients achieving DBP goal (mean DBP <90 mm Hg), the proportion of patients with a mean seated trough cuff SBP reduction of >30 mm Hg, and the proportion of patients with mean seated trough cuff SBP reduction of >40 mm Hg. Other secondary endpoints were described in the primary study publication [12].

The assessment of adverse events (AEs) by risk group was not prespecified but is included here and included serious AEs and those leading to treatment discontinuation, with intensity and causal relationship to the study treatment determined by the investigator.

### 2.4. Statistical Analysis

The objective of these analyses was to investigate whether SPC T80/H25 provided greater BP reductions compared with T80 monotherapy among patient subgroups of DM, renal impairment, and at specific cut-off values for BMI and 10-year risk score for CHD. Efficacy analyses were performed on data from the full analysis set (FAS), defined as randomized patients who received at least one dose of double-blind trial medication, and for whom a baseline measurement and at least one postdose trough efficacy measurement during the high-dose double-blind treatment period were available. Safety analyses were performed on all randomized patients who received at least one dose of the allocated treatment.

The sample size of the trial was calculated to ensure sufficient statistical power to show superiority of SPC T80/H25 over T80 monotherapy with respect to the primary and key secondary endpoints within the overall study population. The subgroup analyses were not powered *per se* to determine the efficacy of SBP H25/T80 versus T80 according to the different patient subpopulations reported here or to test for treatment-by-subgroup interactions. Because this was an exploratory, “proof of concept” analysis and because no adjustments were made to correct for multiplicity, the statistical models were not applied for the purpose of providing statistical significance. *P* values were calculated for the interaction of subgroup and treatment and are considered statistically significant with a *P* value of <0.05. These *P* values provide an indication of whether the differences between treatments differ across subgroup categories (e.g., BMI).

A restricted maximum-likelihood-based, mixed-effect model, and repeated measures approach (using baseline and all available longitudinal observations at each postbaseline visit during the high-dose treatment phase) was utilized for the primary endpoint analysis, as well as for changes from baseline in DBP. This model included the fixed, categorical effects of treatment, country, week, and treatment-by-week interaction, subgroup, and treatment-by-subgroup interaction, with the continuous covariates of baseline mean seated trough cuff SBP or DBP, and baseline-by-week interaction. An unstructured covariance structure was used to model within-patient errors. The difference in least squares means of treatments (SPC T80/H25 versus T80 monotherapy) with a 95% confidence interval (CI) was calculated for each subgroup.

The outcomes of proportion of patients achieving BP, SBP, and DBP goals, and substantial SBP reductions (>30 or >40 mm Hg), were evaluated using logistic regression with fixed effects for treatment, country, subgroup, treatment-by-subgroup interaction, and the respective baseline value (DBP or SBP) as a covariate. Last trough observation carried forward was employed to account for missing data in the analysis of binary endpoints of BP goal achievement and BP reductions. Odds ratios (ORs) with 95% CIs were calculated and reported for the effect of SPC T80/H25 versus T80 monotherapy in different patient subpopulations.

## 3. Results

### 3.1. Patient Characteristics

The baseline characteristics of the entire cohort of 888 patients randomized and treated in the study were previously described [12]. The efficacy analyses were performed on data from 285 patients in the T80 group and 573 patients in the SPC T80/H25 group (FAS total, 858). The safety analyses were performed on data from all 888 treated patients. Compliance with trial medication was high in both treatment groups (at least 96.6% of patients in either treatment group took ≥80% to ≤120% of their trial medication at each visit) [13].

The baseline BP characteristics of different patient subpopulations according to treatment group are shown in Table 1. More patients did not have a diagnosis of DM than those who had a diagnosis with DM (treated set, *n* = 779 and *n* = 109, resp.). More patients had eGFR ≥60 mL/min/1.73 m^2^ (*n* = 824) compared with those with eGFR <60 mL/min/1.73 m^2^ (*n* = 58). Regarding BMI, 188 treated patients had baseline BMI <25 kg/m^2^, 341 treated patients had BMI ≥25–<30 kg/m^2^, and 359 treated patients had BMI ≥30 kg/m^2^. The distribution of patients in low, medium, and high estimated 10-year CHD risk categories is displayed in Table 2. CHD risk was, overall, well matched between the SPC T80/H25 and T80 treatment arms. The majority of patients within this study, 71.3%, were within the lower-risk CHD1 category (<10% risk), 20.9% were in the medium-risk category (≥10%–<20% risk), and 7.8% of patients were within the CHD3 category (≥20% risk).

### 3.2. Efficacy

Efficacy results for the entire study population at Week 7 were previously reported in detail [12]. Briefly, within the entire study population, at Week 7, SPC T80/H25 therapy significantly reduced adjusted mean ± standard error SBP/DBP from baseline (−37.0 ± 0.62/−18.6 ± 0.38 mm Hg) compared with T80 monotherapy (−28.5 ± 0.88/−15.4 ± 0.55 mm Hg (adjusted mean difference −8.5/−3.2 mm Hg; 95% CI, −10.6, −6.4/−4.5, −1.9; *P* < 0.0001)). More patients receiving SPC T80/H25 achieved BP goals compared with patients receiving T80 monotherapy (*P* < 0.0001 for all comparisons).

**Figure 1:**
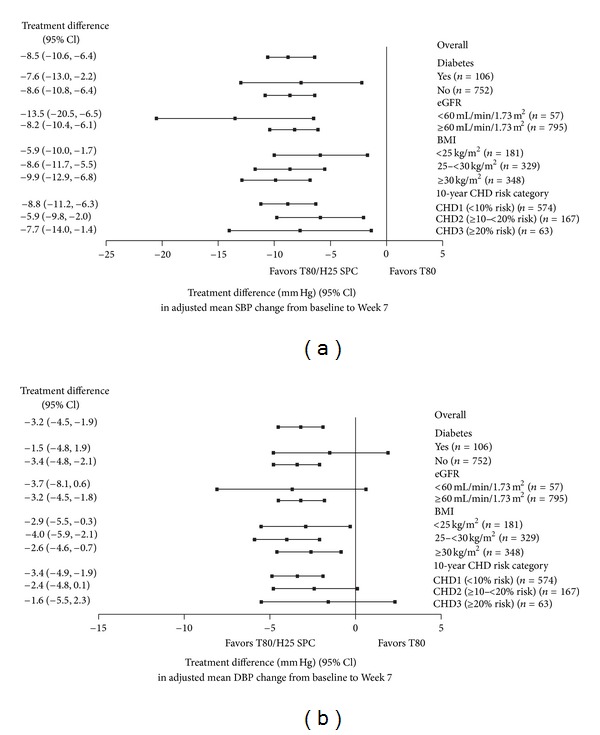
Treatment difference (95% CI) of SPC T80/H25 versus T80 for changes in mean seated trough cuff (a) SBP and (b) DBP from baseline to Week 7 by patient subgroup (FAS). BMI: body mass index; CHD: coronary heart disease; CI: confidence interval; DBP: diastolic blood pressure; eGFR: estimated glomerular filtration rate; FAS: full analysis set; H25: hydrochlorothiazide 25 mg; SBP: systolic blood pressure; SPC: single-pill combination; T80: telmisartan 80 mg.

**Figure 2:**
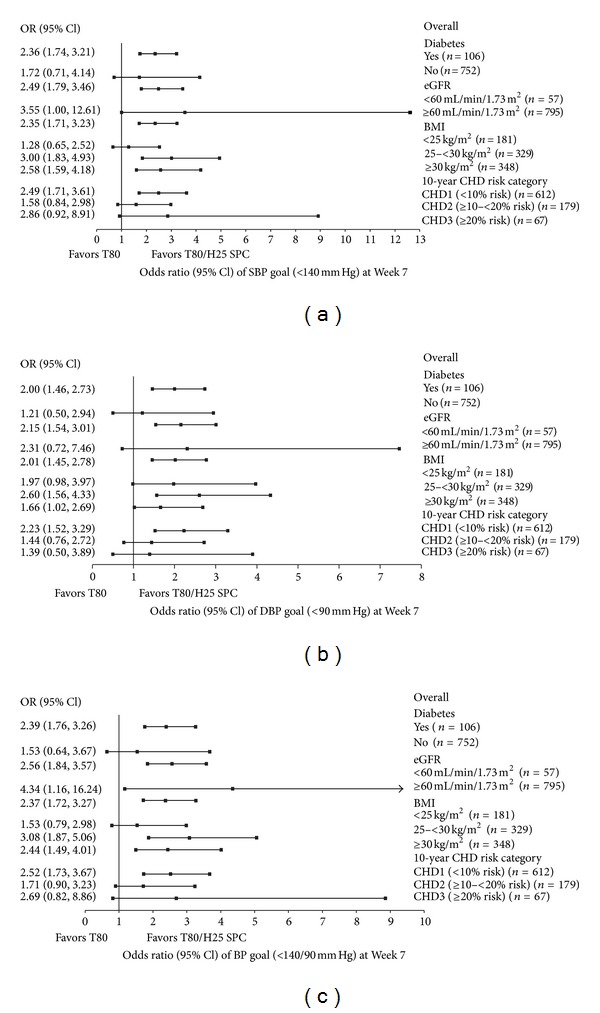
ORs (95% CI) of SPC T80/H25 versus T80 for BP goal rates. (a) SBP (<140 mm Hg), (b) DBP (<90 mm Hg), and (c) SBP/DBP goal (<140/90 mm Hg) at Week 7 by patient subgroup (FAS). BP: blood pressure; BMI: body mass index; CHD: coronary heart disease; CI: confidence interval; DBP: diastolic blood pressure; eGFR: estimated glomerular filtration rate; FAS: full analysis set; H25: hydrochlorothiazide 25 mg; OR: odds ratio; SBP: systolic blood pressure; SPC: single-pill combination; T80: telmisartan 80 mg.

**Figure 3:**
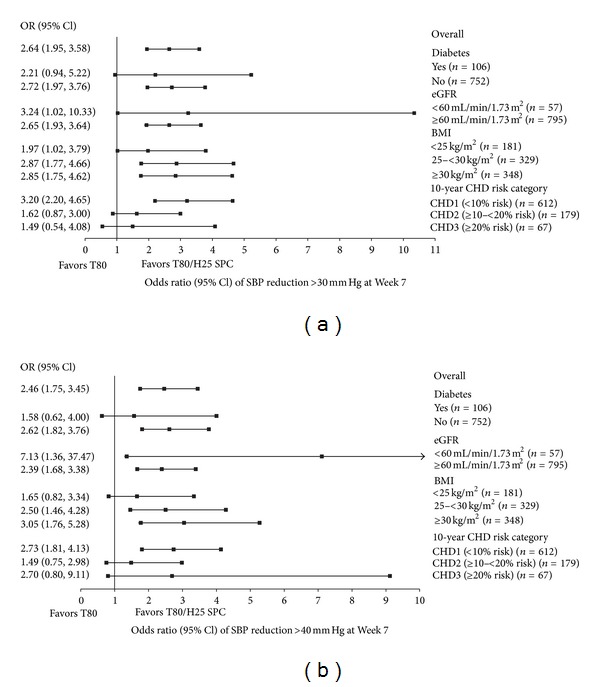
ORs (95% CI) of SPC T80/H25 versus T80 for proportion of patients with seated trough cuff SBP reduction (a) >30 mm Hg and (b) >40 mm Hg at Week 7 by patient subgroup (FAS). BMI: body mass index; CHD, coronary heart disease; CI: confidence interval; eGFR, estimated glomerular filtration rate; FAS: full analysis set; H25: hydrochlorothiazide 25 mg; OR: odds ratio; SBP: systolic blood pressure; SPC, single-pill combination; T80: telmisartan 80 mg.

The observed adjusted mean reductions in SBP/DBP from baseline according to treatment group and patient subpopulations are shown in Table 3. Treatment differences for SPC T80/H25 compared with T80 are depicted in Figure 1. The ORs and 95% CIs for the proportions of patients achieving BP goal and for the proportions of patients achieving SBP reductions >30 or >40 mm Hg, for treatment with SPC T80/H25 versus T80, in the different patient subpopulations, are displayed in Figures 2 and 3, respectively [12]. Descriptions of results according to subpopulations follow.

#### 3.2.1. DM

Only one patient in the SPC T80/H25 treatment group had type 1 DM. No significant treatment by subgroup interactions were found between patients with DM and those without DM with respect to treatment differences in BP reductions and control rates (Table 3 and Figures 1, 2, and 3).

At Week 7, T80/H25 produced greater SBP and DBP reductions compared with T80 monotherapy in patients with or without DM (Table 3 and Figure 1). The proportion of patients with DM who achieved the guideline-based BP goal (SBP/DBP < 130/80 mm Hg) at Week 7 was approximately twofold higher in the SPC T80/H25 group compared with the T80 group (16.7% versus 8.8%, resp.). Among those with DM compared to T80, SPC T80/H25 resulted in a greater percent achievement of the BP goal of <140/90 mm Hg (52.8% versus 38.2%). In patients without DM, this standard BP goal (SBP/DBP < 140/90 mm Hg) was achieved in 55.9% of those patients receiving SPC T80/H25 and 34.3% of those receiving T80.

In patients without DM, greater proportions of patients achieved SBP goal (<140 mm Hg) and DBP goal (<90 mm Hg) with SPC T80/H25 therapy compared to T80 monotherapy (SBP: 64.1% versus 43.0%; DBP: 68.1% versus 51.8%). In patients with DM, SBP and DBP goal rates were also higher in the SPC T80/H25 group compared with the T80 group (SBP: 58.3% versus 41.2%; DBP: 66.7% versus 58.8%) (Figure 2).

In patients without DM, a greater proportion achieved SBP reductions of >30 or >40 mm Hg from baseline to Week  7 with SPC T80/H25 therapy compared with T80 monotherapy (>30 mm Hg: 68.7% versus 46.6%; >40 mm Hg: 41.3% versus 23.1%). In patients with DM, these SBP reductions were also achieved in a higher proportion of patients receiving combination therapy versus T80 monotherapy (>30 mm Hg: 62.5% versus 47.1%; >40 mm Hg: 34.7% versus 29.4%) (Figure 3).

#### 3.2.2. eGFR

No significant treatment-by-subgroup interactions were found between patients within different eGFR categories with respect to treatment differences in BP reductions and control rates (Table 3 and Figures 1, 2, and 3). The large CIs for patients with low eGFR may be related to the low sample size. eGFR is expressed in mL/min/1.73 m².

At Week 7, T80/H25 produced larger SBP and DBP reductions compared with T80 monotherapy in patients within both eGFR categories (Table 3 and Figure 1).

SPC T80/H25 therapy also allowed higher rates of BP goal attainment (<140/90 mm Hg) in patients with eGFR ≥60 (55.7% compared with 35.5% for T80) and in patients with eGFR <60 (52.8% compared with 19.0% for T80).

SBP goal attainment (<140 mm Hg) was also higher in patients receiving SPC T80/H25 compared with T80 monotherapy, regardless of baseline eGFR (eGFR ≥60: 63.8% versus 43.9%; eGFR <60: 55.6% versus 23.8%). The DBP goal of <90 mm Hg for patients with eGFR ≥60 was more commonly achieved in the SPC T80/H25 group (68.5%) compared with the T80 group (53.1%). In patients with eGFR <60, the rates of DBP goal attainment were 58.3% and 42.9%, respectively (Figure 2).

SBP reductions >30 and >40 mm Hg were attained in more of the patients who received SPC T80/H25 compared with T80 monotherapy, regardless of baseline eGFR (>30 mm Hg: 68.7% versus 47.3% in patients with eGFR ≥60; 55.6% versus 33.3% in patients with eGFR <60; >40 mm Hg: 40.9% versus 24.4% in patients with eGFR ≥60; 33.3% versus 9.5% in patients with eGFR <60) (Figure 3).

#### 3.2.3. BMI

No significant treatment-by-subgroup interactions were found between groups of patients within different BMI categories with respect to treatment differences in BP reductions and control rates (Table 3 and Figures 1, 2, and 3). BMI is expressed in kg/m².

Treatment with SPC T80/H25 consistently produced greater reductions in SBP and DBP at Week 7 compared with T80 monotherapy across all three baseline BMI categories (Table 3, Figure 1).

At Week 7, SPC T80/H25 produced higher rates of BP goal attainment (<140/90 mm Hg) versus T80 monotherapy across the three BMI categories (BMI < 25: 58.2% versus 45.8%; BMI 25–<30: 59.3% versus 33.0%; BMI ≥ 30: 50.6% versus 30.6%). The SBP goal (<140 mm Hg) was more commonly achieved in the SPC T80/H25 group compared with the T80 group (BMI < 25: 64.8% versus 55.9%; BMI 25–<30: 65.0% versus 38.3%; BMI ≥ 30: 61.2% versus 40.5%). The DBP goal (<90 mm Hg) was also more commonly achieved in the SPC T80/H25 group compared with the T80 group (BMI < 25: 71.3% versus 57.6%; BMI 25–<30: 73.4% versus 54.8%; BMI ≥ 30: 61.2% versus 47.7%) (Figure 2). A similar pattern was observed for SBP reductions of >30 mm Hg, which were achieved in a greater proportion of patients receiving SPC T80/H25 across all three BMI categories (Figure 3).

#### 3.2.4. 10-*Year* Estimated CHD Risk

The responses of patients within different 10-year CHD risk categories with regard to treatment differences in SBP and DBP reductions are shown in Table 3 for the *post hoc* guideline-driven CHD risk categories and also for the prespecified subgroups of CHD risk divided by tertiles. ORs for treatment differences in SBP/DBP reductions and control rates are displayed in Figures 1, 2, and 3 for the guideline-driven CHD risk categories. No significant treatment-by-subgroup interactions were found.

At Week 7, reductions in mean SBP and DBP were greater in patients receiving SPC T80/H25 compared with T80 monotherapy across all three CHD risk categories (Table 3 and Figure 1).

At Week 7, SPC T80/H25 produced higher rates of BP goal attainment (<140/90 mm Hg) versus T80 monotherapy across the three CHD categories (CHD1: 58.6% versus 36.3%; CHD2: 49.0% versus 37.3%; CHD3: 38.5% versus 17.9%). The SBP goal (<140 mm Hg) was more commonly achieved in the SPC T80/H25 group compared with the T80 group (CHD1: 67.0% versus 45.6%; CHD2: 54.8% versus 44.0%; CHD3: 46.2% versus 21.4%). The DBP goal (<90 mm Hg) was also more commonly achieved in the SPC T80/H25 group compared with the T80 group (CHD1: 69.8% versus 52.2%; CHD2: 62.5% versus 54.7%; CHD3: 61.5% versus 50.0%) (Figure 2). A similar pattern was observed for SBP reductions of >30 mm Hg, which were achieved in a greater proportion of patients receiving SPC T80/H25 across all three CHD categories (Figure 3).

### 3.3. Safety and Tolerability

A summary of AEs reported during the study according to patient subpopulations is provided in Table 4. The proportion of patients experiencing treatment-related AEs in SPC T80/H25 and T80 monotherapy groups was low across most of the patient subpopulations. The proportion of patients with AEs leading to treatment discontinuation was low and comparable across almost all investigated patient subpopulations except the subpopulation of patients with CHD3 (two patients (6.7%) had an AE that resulted in discontinuation within this group). Only one patient experienced a serious AE during the treatment period and that was in the T40 group prior to uptitration. No deaths occurred during the course of the study.

The overall proportion of patients reporting any AE during treatment with SPC T80/H25 was 16.0%. An increase in the rate of AEs was not observed with the presence of DM, increasing BMI, or increasing CHD risk. The frequency of any AE was similar across many of the assessed subpopulations; however, in the subpopulation of patients with DM, the rate was 6.9%, in patients with a low BMI <25 kg/m^2^, the rate was 22.7%, and in patients with a low eGFR <60 mL/min/1.73 m^2^, the rate was 33.3%.

In the overall study population, and irrespective of cause, the five AEs most frequently occurring under treatment with SPC T80/H25, as determined by number of patients receiving SPC T80/H25 with this AE, were dizziness (1.9% of patients), nasopharyngitis (1.4%), pollakiuria (0.9%), vertigo (0.7%), and cough (0.7%). Among patients with DM receiving SPC T80/H25, the most frequently reported AEs, as determined by number of patients with this AE, each occurring in one patient (1.4%), were anal abscess, hypotension, elevated blood uric acid, cough, hypertriglyceridemia, and proctalgia. In patients with eGFR <60 mL/min/1.73 m^2^ who received treatment with SPC T80/H25, the most frequently reported AE was dizziness (*n* = 2 patients, 5.6%). Other AEs occurring in this subpopulation with a frequency of 2.8% (*n* = 1 patient) were upper respiratory tract infection (RTI), nasopharyngitis, ear infection, headache, syncope, hypotension, cough, oropharyngeal pain, abdominal pain, irritable bowel syndrome, myalgia, osteoarthritis, asthenia, and polyp. In patients with BMI ≥ 30 kg/m^2^ who received SPC T80/H25, the most frequent AE was nasopharyngitis (*n* = 4 patients, 1.7%), followed by viral RTI and constipation (each *n* = 3 patients, 1.2%). In high CHD risk patients (CHD3, ≥20%), the most frequent AEs, each with two patients (2.6%), were upper RTI, headache, hiccups, pollakiuria, asthenia, and blood alkaline phosphatase increase.

The overall rate of AEs deemed by the study investigator to be treatment related was 4.6% in the SPC T80/H25 group. A difference in the frequency of treatment-related AEs compared with the overall study population was observed in patients with DM (2.8%), patients with eGFR < 60 mL/min/1.73 m^2^ (16.7%), patients with BMI < 25 kg/m^2^ (7.8%), and patients in the middle CHD risk category CHD2 (6.5%). The most frequent drug-related AEs in the T80/H25 treatment arm were dizziness and pollakiuria (each 0.7%), syncope, and blood uric acid increase (each 0.5%). A breakdown of the frequencies of these four AEs within each subpopulation is provided in Table 5.

## 4. Discussion

The presented results reflect prespecified and *post hoc* subgroup analyses of efficacy and safety data obtained during a 7-week, multinational, phase IV, randomized, double-blind, active-controlled, parallel-group, and forced-titration study in patients with grade 2 or 3 hypertension. In this analysis, SPC T80/H25 similarly reduced SBP and DBP across specific subgroups of patients with CVD risk factors of DM, being overweight/obese, renal impairment, and CHD risk, and those without these CVD risk factors. Additionally, SPC T80/H25 consistently provided greater reductions in SBP and DBP over 7 weeks compared with T80 monotherapy, irrespective of the presence or absence of these CVD risk factors. SPC T80/H25 increased rates of BP goal attainment in all CVD risk groups, including patients with renal impairment, and in overweight to obese patients. Patients with DM were approximately twice as likely to reach a guideline-based BP goal (SBP/DBP < 130/80 mm Hg) over 7 weeks with SPC T80/H25 than with T80 monotherapy (16.7% versus 8.8%), although the sample size of patients with DM was small.

A limitation of this study is that it was designed with a relatively short duration of treatment (7 weeks). Due to the aforementioned reasons, the results obtained from these subgroup analyses should be interpreted under consideration of their exploratory character. However, this is the first report on the efficacy of SPC T80/H25 in specific patient subpopulations according to the presence or absence of CVD risk factors (DM, being overweight/obese, renal impairment, and high CHD risk).

BP control is important in all hypertensive patients but is especially pertinent in those with additional CVD risk factors, since elevated BP is associated with significant increases in CV risk [16]. It is recognized that combination therapy can reduce BP to a greater extent and achieve BP goals more promptly [6], so high-risk individuals are likely to benefit from protective interventions without any delay. Initial combination therapy is increasingly recommended by guidelines, particularly for patients with CVD risk factors [6], and well-tolerated combinations can facilitate improved treatment adherence, a key factor in achieving successful BP control. Outcome studies have demonstrated that for every 20 mm Hg reduction in SBP, there is a 40–50% reduction in CVD [17]. The degree of BP reductions observed with SPC T80/H25 during this study would be expected to reduce CVD morbidity and mortality among patients at high risk for CVD [5, 6]. These analyses indicate that SPC T80/H25 is an effective and generally well-tolerated antihypertensive combination that is suitable for treating a wide range of patients with grade 2 or 3 hypertension, in the presence or absence of additional CVD risk factors.

## 5. Conclusions

These analyses indicate that in patients with grade 2 or 3 hypertension, SPC T80/H25 consistently provided greater BP reductions and increased attainment of BP goals compared with T80 monotherapy among patient subgroups with CVD risk factors. No consistent pattern of differences in AEs was seen in this short trial. The majority of patients with hypertension will require combination therapy to reach BP goals [5, 18]. The results of this trial indicate that treatment with SPC T80/H25 in patients with grade 2 or 3 hypertension provides greater BP reductions and higher rates of goal attainment compared with T80 monotherapy. Antihypertensive treatment with the T80/H25 single-pill combination is effective and generally well tolerated, irrespective of the presence of additional CVD risk factors.

## Figures and Tables

**Table 1:** Baseline characteristics in the different patient subpopulations, based on treated patients.

	T40/T80	T40/H12.5/T80/H25
DM*, yes	(*n* = 35)	(*n* = 74)
Gender	22 male/13 female	38 male/36 female
Age, mean ± SD	61.8 ± 8.6	61.1 ± 9.2
BMI, mean ± SD	30.7 ± 5.6	31.1 ± 5.3
SBP/DBP (mm Hg), mean ± SD^a^	174.0 ± 9.7/103.6 ± 4.6	171.1 ± 8.4/103.3 ± 3.7
Range SBP/DBP (mm Hg)^a^	163–193/97–118	156–195/94–112
DM*, no	(*n* = 259)	(*n* = 520)
Gender	147 male/112 female	271 male/249 female
Age, mean ± SD	57.0 ± 12.1	56.2 ± 11.4
BMI, mean ± SD	28.9 ± 5.6	29.4 ± 5.4
SBP/DBP (mm Hg), mean ± SD^a^	173.1 ± 9.3/104.6 ± 5.0	172.4 ± 9.8/104.4 ± 5.0
Range SBP/DBP (mm Hg)^a^	155–197/92–119	131–211/87–119

eGFR < 60 mL/min/1.73 m^2^	(*n* = 21)	(*n* = 37)
Gender	10 male/11 female	18 male/19 female
Age, mean ± SD	70.1 ± 9.1	65.4 ± 9.4
BMI, mean ± SD	28.3 ± 5.0	30.7 ± 5.4
SBP/DBP (mm Hg), mean ± SD^b^	177.7 ± 10.8/103.4 ± 5.9	173.1 ± 9.6/104.2 ± 5.0
Range SBP/DBP (mm Hg)^b^	161–195/96–119	160–193/97–118
eGFR ≥ 60 mL/min/1.73 m^2^	(*n* = 271)	(*n* = 553)
Gender	159 male/112 female	288 male/265 female
Age, mean ± SD	56.6 ± 11.5	56.2 ± 11.1
BMI, mean ± SD	29.1 ± 5.4	29.5 ± 5.4
SBP/DBP (mm Hg), mean ± SD^b^	172.8 ± 9.2/104.6 ± 4.9	172.2 ± 9.7/104.2 ± 4.9
Range SBP/DBP (mm Hg)^b^	155–197/92–118	131–211/87–119

BMI < 25 kg/m^2^	(*n* = 60)	(*n* = 128)
Gender	38 male/22 female	69 male/59 female
Age, mean ± SD	57.6 ± 11.9	57.3 ± 13.4
BMI, mean ± SD	22.6 ± 2.0	23.2 ± 1.5
SBP/DBP (mm Hg), mean ± SD^c^	172.5 ± 10.0/103.9 ± 4.9	170.3 ± 9.6/103.7 ± 4.5
Range SBP/DBP (mm Hg)^c^	160–195/96–117	160–198/95–119
BMI 25–<30 kg/m^2^	(*n* = 120)	(*n* = 221)
Gender	68 male/52 female	125 male/96 female
Age, mean ± SD	59.6 ± 10.7	57.5 ± 10.1
BMI, mean ± SD	27.2 ± 1.5	27.5 ± 1.4
SBP/DBP (mm Hg), mean ± SD^c^	173.3 ± 8.7/103.9 ± 4.7	171.9 ± 9.6/104.1 ± 4.7
Range SBP/DBP (mm Hg)^c^	160–197/92–118	131–195/87–119
BMI ≥ 30 kg/m^2^	(*n* = 114)	(*n* = 245)
Gender	63 male/51 female	115 male/130 female
Age, mean ± SD	55.4 ± 12.7	55.9 ± 11.0
BMI, mean ± SD	34.6 ± 4.5	34.8 ± 4.1
SBP/DBP (mm Hg), mean ± SD^c^	173.4 ± 9.7/105.3 ± 5.1	173.6 ± 9.6/104.6 ± 5.1
Range SBP/DBP (mm Hg)^c^	155–194/95–119	143–211/91–119

CHD1 10-year risk <10%	(*n* = 187)	(*n* = 446)
Gender	63 male/124 female	164 male/282 female
Age, mean ± SD	55.3 ± 12.5	55.4 ± 11.7
BMI, mean ± SD	29.7 ± 6.2	29.8 ± 5.7
SBP/DBP (mm Hg), mean ± SD^d^	172.9 ± 9.6/104.7 ± 5.3	172.0 ± 9.7/104.4 ± 5.0
Range^a^ SBP/DBP (mm Hg)^d^	155–197/95–119	131–211/87–119
CHD2 10-year risk ≥10–<20%	(*n* = 77)	(*n* = 109)
Gender	76 male/1 female	106 male/3 female
Age, mean ± SD	59.6 ± 9.5	59.3 ± 8.0
BMI, mean ± SD	27.8 ± 4.1	28.4 ± 4.1
SBP/DBP (mm Hg), mean ± SD^d^	172.3 ± 9.3/104.0 ± 4.3	172.4 ± 9.5/104.0 ± 4.2
Range SBP/DBP (mm Hg)^d^	160–190/92–113	160–198/95–115
CHD3 10-year risk ≥20%	(*n* = 30)	(*n* = 39)
Gender	30 male/0 female	39 male/0 female
Age, mean ± SD	66.5 ± 7.1	64.8 ± 9.0
BMI, mean ± SD	29.3 ± 4.5	30.0 ± 4.5
SBP/DBP (mm Hg), mean ± SD^d^	176.9 ± 7.3/104.2 ± 4.8	174.7 ± 10.2/102.7 ± 3.9
Range SBP/DBP (mm Hg)^d^	164–191/97–118	156–195/94–115

CHD risk tertile 1 (<3.62%)	(*n* = 92 )	(*n* = 202)
Gender	11 male/81 female	26 male/176 female
Age, mean ± SD	55.8 ± 14.5	55.6 ± 14.2
BMI, mean ± SD	30.0 ± 6.5	29.6 ± 6.2
SBP/DBP (mm Hg), mean ± SD^e^	172.2 ± 9.8/104.8 ± 5.0	172.1 ± 10.3/103.9 ± 4.8
Range^a^ SBP/DBP (mm Hg)^e^	155–194/95–118	131–211/87–119
CHD risk tertile 2 (≥3.62–<8.66%)	(*n* = 83)	(*n* = 212)
Gender	43 male/40 female	108 male/104 female
Age, mean ± SD	54.4 ± 10.1	55.2 ± 9.4
BMI, mean ± SD	29.1 ± 5.9	30.2 ± 5.3
SBP/DBP (mm Hg), mean ± SD^e^	174.2 ± 9.4/105.1 ± 5.6	171.7 ± 9.2/105.0 ± 5.2
Range SBP/DBP (mm Hg)^e^	160–197/95–119	143–194/91–119
CHD risk tertile 3 (≥8.66%)	(*n* = 119)	(*n* = 180)
Gender	115 male/4 female	175 male/5 female
Age, mean ± SD	61.1 ± 9.6	59.9 ± 8.8
BMI, mean ± SD	28.5 ± 4.5	28.9 ± 4.5
SBP/DBP (mm Hg), mean ± SD^e^	173.2 ± 9.0/103.8 ± 4.3	173.1 ± 9.5/103.7 ± 4.3
Range SBP/DBP (mm Hg)^e^	160–191/92–118	156–198/94–117

*Based on the presence of type 1 DM and type 2 DM, presence of diabetic retinopathy or diabetic nephropathy, and recognized MedDRA codes for diabetes.

SBP/DBP data from FAS (*n*):

^a^DM (yes) FAS: T40/T80, *n* = 34; T40/H12.5/T80/H25, *n* = 72; DM (no) FAS: T40/T80, *n* = 251; T40/H12.5/T80/H25, *n* = 501.

^b^eGFR < 60 FAS: T40/T80, *n* = 21; T40/H12.5/T80/H25, *n* = 36; eGFR ≥ 60 FAS: T40/T80, *n* = 262; T40/H12.5/T80/H25, *n* = 533.

^c^BMI < 25 FAS: T40/T80, *n* = 59; T40/H12.5/T80/H25, *n* = 122; BMI 25–<30 FAS: T40/T80, *n* = 115; T40/H12.5/T80/H25, *n* = 214; BMI ≥ 30 FAS: T40/T80, *n* = 111; T40/H12.5/T80/H25, *n* = 237.

^d^CHD1 FAS: T40/T80, *n* = 182; T40/H12.5/T80/H25, *n* = 430; CHD2 FAS: T40/T80, *n* = 75; T40/H12.5/T80/H25, *n* = 104; CHD3 FAS: T40/T80, *n* = 28; T40/H12.5/T80/H25, *n* = 39.

^e^CHD tertile 1 FAS: T40/T80, *n* = 90; T40/H12.5/T80/H25, *n* = 194; CHD tertile 2 FAS: T40/T80, *n* = 81; T40/H12.5/T80/H25, *n* = 205; CHD tertile 3 FAS: T40/T80, *n* = 114; T40/H12.5/T80/H25, *n* = 174.

BMI: body mass index; CHD: coronary heart disease; DBP: diastolic blood pressure; DM: diabetes mellitus; eGFR: estimated glomerular filtration rate; FAS: full analysis set; H12.5: hydrochlorothiazide 12.5 mg; H25: hydrochlorothiazide 25 mg; MedDRA: Medical Dictionary for Regulatory Activities; SBP: systolic blood pressure; SD: standard deviation; T40: telmisartan 40 mg; T80: telmisartan 80 mg.

**Table 2:** Probability of developing CHD over 10 years: descriptive statistics and distribution of patients within estimated 10-year CHD risk categories, based on treated patients.

	T40/T80	T40/H12.5/T80/H25	Total
Total patients (*N*)	294	594	888
Overall probability of developing CHD over 10 years			
Mean (SD)	9.06 (7.89)	7.66 (7.12)	8.12 (7.41)
Range (min–max)	0.35–51.06	0.04–49.39	0.04–51.06
Patients in CHD risk category, *N* (%)			
CHD 1 (<10%)	187 (63.6)	446 (75.1)	633 (71.3)
CHD 2 (10–<20% )	77 (26.2)	109 (18.4)	186 (20.9)
CHD 3 (≥20%)	30 (10.2)	39 (6.6)	69 (7.8)

Percentages may not sum to 100% due to rounding.

CHD: coronary heart disease; H12.5: hydrochlorothiazide 12.5 mg; H25: hydrochlorothiazide 25 mg; SD: standard deviation; T40: telmisartan 40 mg; T80: telmisartan 80 mg.

**Table 3:** SBP and DBP reductions (mm Hg) from baseline to Week 7 in overall patient population and different patient subgroups, based on the FAS.

	Adjusted mean SBP/DBP reduction from baseline		Adjusted mean treatment difference of T80/H25 versus T80 (95% CI)	
	T80	T80/H25	SBP	DBP
Overall population	−28.5/−15.4	−37.0/−18.6	−8.5 (−10.6, −6.4)	−3.2 (−4.5, −1.9)
DM				
Yes	−26.7/−17.2	−34.2/−18.6	−7.6 (−13.0, −2.2)	−1.5 (−4.8, 1.9)
No	−28.7/−15.1	−37.4/−18.6	−8.6 (−10.8, −6.4)	−3.4 (−4.8, −2.1)
eGFR category				
<60 mL/min/1.73 m^2^	−20.3/−13.3	−33.8/−17.0	−13.5 (−20.5, −6.5)	−3.7 ( −8.1, 0.6)
≥60 mL/min/1.73 m^2^	−28.9/−15.5	−37.2/−18.7	−8.2 (−10.4, −6.1)	−3.2 (−4.5, −1.8)
BMI category				
<25 kg/m^2^	−30.1/−16.6	−36.0/−19.5	−5.9 (−10.0, −1.7)	−2.9 (−5.5, −0.3)
25–<30 kg/m^2^	−28.9/−14.9	−37.5/−18.9	−8.6 (−11.7, −5.5)	−4.0 (−5.9, −2.1)
≥30 kg/m^2^	−27.1/−15.2	−37.0/−17.8	−9.9 (−12.9, −6.8)	−2.6 (−4.6, −0.7)
10-year CHD risk category				
CHD1 (risk < 10%)	−29.5/−15.7	−38.3/−19.1	−8.8 (−11.2, −6.3)	−3.4 (−4.9, −1.9)
CHD2 (risk ≥ 10–<20%)	−27.2/−14.9	−33.1/−17.3	−5.9 (−9.8, −2.0)	−2.4 (−4.8, 0.1)
CHD3 (risk ≥ 20%)	−25.3/−14.7	−32.9/−16.3	−7.7 (−14.0, −1.4)	−1.6 (−5.5, 2.3)
10-year CHD risk by tertiles				
CHD risk tertile 1 (<3.62%)	−31.1/−17.9	−38.9/−19.6	−7.8 (−11.2, −4.5)	−1.7 (−3.8, 0.4)
CHD risk tertile 2 (≥3.62–<8.66%)	−27.4/−13.4	−37.7/−18.9	−10.3 (−13.8, −6.9)	−5.5 (−7.6, −3.3)
CHD risk tertile 3 (≥8.66%)	−27.2/−14.9	−33.9/−17.2	−6.7 (−9.9, −3.6)	−2.4 (−4.3, −0.4)

BMI: body mass index; CHD: coronary heart disease; CI: confidence interval; DBP: diastolic blood pressure; DM: diabetes mellitus; eGFR: estimated glomerular filtration rate; FAS: full analysis set; H25: hydrochlorothiazide 25 mg; SBP: systolic blood pressure; T80: telmisartan 80 mg.

**Table 4:** Summary of AE frequencies in the different patient subpopulations, based on treated patients.

	T80	T80/H25
Overall	(*n* = 289)	(*n* = 586)
Patients with any AE, *N* (%)	49 (17.0)	94 (16.0)
Patients with treatment-related AEs, *N* (%)	8 (2.8)	27 (4.6)
Patients with AEs leading to discontinuation, *N* (%)	8 (2.8)	6 (1.0)
Patients with serious AEs, *N* (%)	0 (0)	0 (0)

DM, yes	(*n* = 34)	(*n* = 72)
Patients with any AE, *N* (%)	6 (17.6)	5 (6.9)
Patients with treatment-related AEs, *N* (%)	1 (2.9)	2 (2.8)
Patients with AEs leading to discontinuation, *N* (%)	0 (0)	0 (0)
Patients with serious AEs, *N* (%)	0 (0)	0 (0)
DM, no	(*n* = 255)	(*n* = 514)
Patients with any AE, *N* (%)	43 (16.9)	89 (17.3)
Patients with treatment-related AEs, *N* (%)	7 (2.7)	25 (4.9)
Patients with AEs leading to discontinuation, *N* (%)	8 (3.1)	6 (1.2)
Patients with serious AEs, *N* (%)	0 (0)	0 (0)

eGFR category <60 mL/min/1.73 m^2^	(*n* = 21)	(*n* = 36)
Patients with any AE, *N* (%)	4 (19.0)	12 (33.3)
Patients with treatment-related AEs, *N* (%)	0 (0)	6 (16.7)
Patients with AEs leading to discontinuation, *N* (%)	0 (0)	1 (2.8)
Patients with serious AEs, *N* (%)	0 (0)	0 (0)
eGFR category ≥60 mL/min/1.73 m^2^	(*n* = 266)	(*n* = 546)
Patients with any AE, *N* (%)	45 (16.9)	81 (14.8)
Patients with treatment-related AEs, *N* (%)	8 (3.0)	21 (3.8)
Patients with AEs leading to discontinuation, *N* (%)	8 (3.0)	5 (0.9)
Patients with serious AEs, *N* (%)	0 (0)	0 (0)

BMI < 25 kg/m^2^	(*n* = 59)	(*n* = 128)
Patients with any AE, *N* (%)	8 (13.6)	29 (22.7)
Patients with treatment-related AEs, *N* (%)	2 (3.4)	10 (7.8)
Patients with AEs leading to discontinuation, *N* (%)	2 (3.4)	3 (2.3)
Patients with serious AEs, *N* (%)	0 (0)	0 (0)
BMI 25–<30 kg/m^2^	(*n* = 118)	(*n* = 217)
Patients with any AE, *N* (%)	22 (18.6)	25 (11.5)
Patients with treatment-related AEs, *N* (%)	4 (3.4)	7 (3.2)
Patients with AEs leading to discontinuation, *N* (%)	3 (2.5)	2 (0.9)
Patients with serious AEs, *N* (%)	0 (0)	0 (0)
BMI ≥ 30 kg/m^2^	(*n* = 112)	(*n* = 241)
Patients with any AE, *N* (%)	19 (17.0)	40 (16.6)
Patients with treatment-related AEs, *N* (%)	2 (1.8)	10 (4.1)
Patients with AEs leading to discontinuation, *N* (%)	3 (2.7)	1 (0.4)
Patients with serious AEs, *N* (%)	0 (0)	0 (0)

CHD1 (10-year risk <10%)	(*n* = 183)	(*n* = 439)
Patients with any AE, *N* (%)	35 (19.1)	72 (16.4)
Patients with treatment-related AEs, *N* (%)	4 (2.2)	18 (4.1)
Patients with AEs leading to discontinuation, *N* (%)	4 (2.2)	5 (1.1)
Patients with serious AEs, *N* (%)	0 (0)	0 (0)
CHD2 (10-year risk ≥10–<20%)	(*n* = 76)	(*n* = 108)
Patients with any AE, *N* (%)	10 (13.2)	17 (15.7)
Patients with treatment-related AEs, *N* (%)	2 (2.6)	7 (6.5)
Patients with AEs leading to discontinuation, *N* (%)	2 (2.6)	1 (0.9)
Patients with serious AEs, *N* (%)	0 (0)	0 (0)
CHD3 (10-year risk ≥20%)	(*n* = 30)	(*n* = 39)
Patients with any AE, *N* (%)	4 (13.3)	5 (12.8)
Patients with treatment-related AEs, *N* (%)	2 (6.7)	2 (5.1)
Patients with AEs leading to discontinuation, *N* (%)	2 (6.7)	0 (0)
Patients with serious AEs, *N* (%)	0 (0)	0 (0)

AE: adverse event; BMI: body mass index; CHD: coronary heart disease; DM: diabetes mellitus; eGFR: estimated glomerular filtration rate; H25: hydrochlorothiazide 25 mg; T80: telmisartan 80 mg.

**Table 5:** Frequency of the most commonly observed treatment-related AEs occurring in patients receiving SPC T80/H25 treatment: by treatment group and patient subpopulation; *N* (%): based on treated patients.

	Total *N* in subgroup		Dizziness		Syncope		Pollakiuria		Blood uric acid increased	
	T80	T80/H25	T80	T80/H25	T80	T80/H25	T80	T80/H25	T80	T80/H25
Overall	**289**	**586**	3 (1.0)	11 (1.9)	0 (0)	3 (0.5)	0 (0)	5 (0.9)	0 (0)	3 (0.5)

DM, yes	**34**	**72**	0 (0)	0 (0)	0 (0)	0 (0)	0 (0)	0 (0)	0 (0)	1 (1.4)
DM, no	**43**	**89**	3 (1.2)	11 (2.1)	0 (0)	3 (0.6)	0 (0)	5 (1.0)	0 (0)	2 (0.4)

BMI < 25	**59**	**128**	0 (0)	5 (3.9)	0 (0)	0 (0)	0 (0)	2 (1.6)	0 (0)	1 (0.8)
BMI 25–<30	**118**	**217**	1 (0.8)	4 (1.8)	0 (0)	1 (0.5)	0 (0)	1 (0.5)	0 (0)	2 (0.9)
BMI ≥ 30	**112**	**241**	2 (1.8)	2 (0.8)	0 (0)	2 (0.8)	0 (0)	2 (0.8)	0 (0)	0 (0)

eGFR < 60	**21**	**36**	0 (0)	2 (5.6)	0 (0)	1 (2.8)	0 (0)	0 (0)	0 (0)	0 (0)
eGFR ≥ 60	**266**	**546**	3 (1.1)	9 (1.6)	0 (0)	2 (0.4)	0 (0)	5 (0.9)	0 (0)	3 (0.5)

CHD1	**183**	**439**	3 (1.6)	10 (2.3)	0 (0)	2 (0.5)	0 (0)	2 (0.5)	0 (0)	1 (0.2)
CHD2	**76**	**108**	0 (0)	1 (0.9)	0 (0)	1 (0.9)	0 (0)	2 (1.9)	0 (0)	2 (1.9)
CHD3	**30**	**39**	0 (0)	0 (0)	0 (0)	0 (0)	0 (0)	1 (2.6)	0 (0)	0 (0)

Data based on treated set. eGFR expressed in mL/min/1.73 m^2^; BMI expressed in kg/m^2^.

BMI: body mass index; CHD: coronary heart disease; DM: diabetes mellitus; eGFR: estimated glomerular filtration rate; H25: hydrochlorothiazide 25 mg; T80: telmisartan 80 mg.
